# Ultrasound-Guided Quadratus Lumborum Block Enhances the Quality of Recovery after Gastrointestinal Surgery: A Randomized Controlled Trial

**DOI:** 10.1155/2022/8994297

**Published:** 2022-04-30

**Authors:** Qing-Ren Liu, Yu-Chen Dai, Jue Xie, Xiang Li, Xing-Bing Sun, Jie Sun

**Affiliations:** ^1^Department of Anesthesiology, Xishan People's Hospital of Wuxi City, Wuxi 214105, China; ^2^Department of Anesthesiology, Zhongda Hospital, Medical School, Southeast University, Nanjing 210009, China

## Abstract

**Background:**

Quadratus lumborum block (QLB) has been used to reduce postoperative acute pain and opioid consumption. However, the efficacy of QLB on the quality of recovery (QoR) after gastrointestinal surgery has not been established. The aim of this study was to evaluate the ability of QLB to enhance the postoperative QoR in patients undergoing open gastrointestinal surgery.

**Methods:**

Eighty-four patients undergoing open gastrointestinal surgery were randomized to receive ultrasound-guided QLB with either 20 ml of 0.375% ropivacaine or saline. The primary outcome was the QoR-15 score at 24 h after surgery. The secondary outcomes were the postoperative pain intensity, opioid consumption, the incidence of nausea, vomiting, and chronic pain.

**Results:**

The global QoR-15 score at 24 h postoperatively was significantly higher in the QLB group than in the control group (mean difference: 16.9; 95% CI: 11.9–21.9). Additionally, the QoR-15 scores for five dimensions were significantly higher in the QLB group than in the control group. The cumulative oxycodone consumption was significantly lower in the QLB group during 0–6, 6–24, 0–24, 24–48, and 0–48 h postoperatively than in the control group. At rest or during coughing, the pain verbal rating scale scores were significantly lower at 1, 3, 6, 12, and 24 h after surgery in the QLB group than in the control group. The incidence of postoperative nausea was significantly different between the groups, but postoperative vomiting was not.

**Conclusion:**

Single-injection posteromedial QLB with ropivacaine enhanced the QoR at 48 h after surgery and improved analgesia during the early postoperative period in patients undergoing gastrointestinal surgery.

## 1. Introduction

Gastric and colorectal cancer is the most common malignancy and is strongly associated with high mortality [1]. Open and laparoscopic radical resection is the standard surgical treatment for gastric and colorectal cancer. Although laparoscopic surgery is less invasive, it can still generate moderate to severe acute postoperative pain [2], which may significantly influence the quality of recovery (QoR) [3].

Regional analgesic techniques, as an integral component of rapid recovery after gastrointestinal surgery, have been used to control pain in recent decades. However, the effects of these approaches remain controversial. It is well-known that epidural anesthesia effectively provides superior postoperative analgesia and QoR [4], but it is limited because of the complexity of the operation and the high incidence of hypotension. Limited efficacy was also observed in the transversus abdominis plane (TAP) block due to its narrow coverage and short duration, although it can reduce postoperative pain and improve QoR [5, 6]. In addition, TAP block cannot effectively relieve visceral pain [7, 8]. Therefore, it is critical to developing a more effective regional analgesic technique for patients undergoing abdominal surgery.

Quadratus lumborum block (QLB), as a recently discovered truncal regional block technique, can effectively reduce postoperative pain and opioid consumption for total hip arthroplasty [9, 10], inguinal hernia repair [11, 12], cholecystectomy [13, 14], caesarean section [8, 15], gynecologic surgery [16], percutaneous nephrolithotomy [17], nephrectomy [18, 19], and colorectal surgery [20, 21]. Furthermore, QLB improves the postoperative QoR in total hip arthroplasty [22] and laparoscopic partial nephrectomy [23]. The QoR is an index of the health status of patients after surgery, which includes five dimensions of health, namely, physical comfort, physical independence, psychological support, emotional status, and pain. The 15-item quality of recovery (QoR-15) scale, a simplified version of the 40-item quality of recovery (QoR-40) scale, which is presented as a score ranging from 0 to 150, with 150 corresponding to the best possible outcome, has been widely used in patients undergoing different operations and anesthetic techniques [24–27]. Furthermore, high-quality evidence for good content validity, internal consistency, and unidimensionality of the QoR-15 was found in different languages [28–30].

To date, no randomized controlled trials have reported the efficacy of QLB for improving the QoR scores after gastrointestinal surgery. Ökmen et al. [13] reported that posterior QLB provided superior analgesia in patients who underwent laparoscopic cholecystectomy. In this prospective trial, we implemented posteromedial QLB, in which local anesthetics were deposited deep into the posterior thoracolumbar fascia between the quadratus lumborum and the erector spinae. We hypothesized that posteromedial QLB could not only enhance the QoR after gastrointestinal surgery but also reduce postoperative pain intensity, opioid consumption, adverse effects, and intraoperative remifentanil consumption.

## 2. Methods

### 2.1. Study Design

This prospective, double-blinded, randomized controlled trial was conducted at Xishan People's Hospital of Wuxi City in China between August 2019 and January 2020. Ethical approval for this study (No. 2019ZDSYLL084-P01) was provided by the Independent Ethics Committee for Clinical Research of Xishan People's Hospital of Wuxi City (chairperson: Professor Jun Gu) on 18 July 2019. The protocol was registered at the Chinese Clinical Trial Registry (ChiCTR1900024865). Written informed consent was obtained as a condition for participating in the study, and Consolidated Standards of Reporting Trials (CONSORT) guidelines were followed.

### 2.2. Participants

Patients of the American Society of Anesthesiologists physical status I to III, aged 18 to 80 years and scheduled for elective gastrointestinal surgery due to gastric or colorectal cancer, were enrolled in the study. The exclusion criteria were as follows: evidence of coagulation dysfunction, a body mass index (BMI) less than 15 kg/m^2^ or more than 30 kg/m^2^, infection at the puncture site, a history of severe heart or lung disease, hepatic and renal insufficiency, mental illness, and self-expression disorders. Participants were randomly allocated into the QLB combined with the general anesthesia group (QLB group) or the general anesthesia group (control group) in a 1:1 ratio. Randomization was performed using a computerized random number generator (https://www.randomization.com). The group allocation numbers were concealed in sequentially numbered envelopes. All participants and investigators were blinded to the group assignment.

### 2.3. Quadratus Lumborum Block

Prior to the induction of anesthesia, single-injection QLB was performed by an anesthesiologist familiar with ultrasound-guided nerve blocks. To identify the quadratus lumborum, psoas, erector spinae, and transverse process, a curvilinear probe (2–6 MHz) was placed at the posterior axillary line between the costal margin and the iliac crest. A 22-gauge, 100 mm needle tip was advanced from the posterolateral side using an in-plane technique, aiming to reach the lumbar interfascial triangle on the medial edge of the quadratus lumborum, which is located between the quadratus lumborum and the erector spinae muscle. Subsequently, 20 mL of 0.375% ropivacaine or saline was injected into the thoracolumbar fascia bilaterally once the needle tip was determined.

### 2.4. Anesthetic and Analgesic Techniques

Preoxygenation and standard monitoring were performed after patients were transported to the operating room, without premedication. For anesthesia induction, sufentanil (0.3 *μ*g/kg), propofol (2–2.5 mg/kg), and rocuronium (0.6 mg/kg) were administered. Anesthesia was maintained with sevoflurane (minimum alveolar concentration, 0.7–1.0), remifentanil (0.05–0.2 *μ*g/kg/min), and cisatracurium 5 mg intermittently. All gastrointestinal cancer operations were performed by the same group of surgeons, which included four surgeons. Surgical technique and extent of surgery followed with the standardization of surgery as described in previous consensus guidelines for the management of gastric and colorectal cancers. During skin closure, sufentanil (0.1 mg/kg) and ondansetron (0.1 mg/kg) were given intravenously for analgesia and antiemetic prophylaxis, respectively. All patients were transferred to the postanesthesia care unit (PACU) after surgery, where the neuromuscular blockade was antagonized with neostigmine and atropine. When patients were fully awake and muscle tone was fully restored, extubation was performed.

For standard postoperative pain management, patients received 2 mg of oxycodone injection when the pain verbal rating scale (VRS) score at rest was ˃3 in the PACU. In addition, 200 mg of oral celecoxib was administered every 12 h, and patient-controlled intravenous analgesia (0.5 mg/mL oxycodone, without background infusion; bolus 4 ml; lock time of 5 min) was performed for 48 h postoperatively on the ward.

### 2.5. Outcome Measurements

The primary outcome was the global QoR-15 score at 24 h after surgery. The QoR-15 score comprises 15 questions that assess 5 recovery domains, namely, physical comfort, physical independence, psychological support, emotional status, and pain [24, 31]. Each question is scored from 0 to 10 (0 = none of the time to 10 = all of the time). All patients completed the questionnaire 1 day before surgery and 24 and 48 h after surgery.

The secondary outcomes included postoperative pain, cumulative oxycodone consumption, adverse effects, and intraoperative remifentanil consumption. The cumulative oxycodone consumption during 0–6, 6–24, 0–24, 24–48, and 0–48 h after surgery was collected. The pain intensity was assessed using the VRS, which is a reliable and easily understood tool for the assessment of pain [32]. The pain scores at rest and during coughing were recorded at 1, 3, 6, 12, 24, and 48 h after surgery. Adverse effects including postoperative nausea vomiting, dizziness, and pruritus were also recorded. All patients were asked to assess the occurrence of chronic pain at the surgical site using the numeric rating scale (NRS) score by phone at 3 and 6 months after surgery. CPSP was defined as the pain VRS score at rest ≥1.

On the day before surgery, demographic characteristics including sex, age, height, weight, and underlying medical conditions such as hypertension and diabetes were collected. Patients were asked to rate their average expected pain intensity on the first day after surgery (on a scale from 0 = no pain at all to 10 = strongest pain imaginable) [33]. The preoperative state of anxiety and depression was assessed by the hospital anxiety and depression scale [34]. The following data were also collected: remifentanil consumption during the operation, blood loss volume, duration of anesthesia, duration of surgery, length of hospital stay, indwelling tube time, and time to leave the bed (time to the first getting out of bed after surgery).

### 2.6. Statistical Analysis

The sample size was calculated based on the global QoR-15 score. In our preliminary study, the mean and standard deviation (SD) of global QoR-15 scores were estimated to be 115.7 and 14.2, respectively, at 24 h after surgery. A change of 8 for the QoR-15 scores was considered to represent a clinically relevant difference [35, 36]. Using the two-sample *t*-test, 39 patients per group would be required at a significance level of 0.05 and a power of 0.8. After considering the possible dropout rate of 15%, we enrolled 46 patients per group.

IBM SPSS software version 26.0 (IBM, Armonk, NY, USA) was used to analyze the data. Continuous variables are presented as the mean ± SD or median (IQR), and categorical variables are presented as numbers (percentages). The assumptions of normality for continuous variables were assessed using the Shapiro–Wilk test and Q-Q plot. Between-group differences were evaluated using the independent sample *t*-test or Mann–Whitney *U* test for continuous parametric variables or nonparametric variables. Categorical variables were compared between groups using the chi-square test or Fisher's exact test. Repeated measures analysis of variance was used for time-series data such as the pain NRS score. A *P* value of <0.05 was considered statistically significant.

## 3. Results

**Figure 1:**
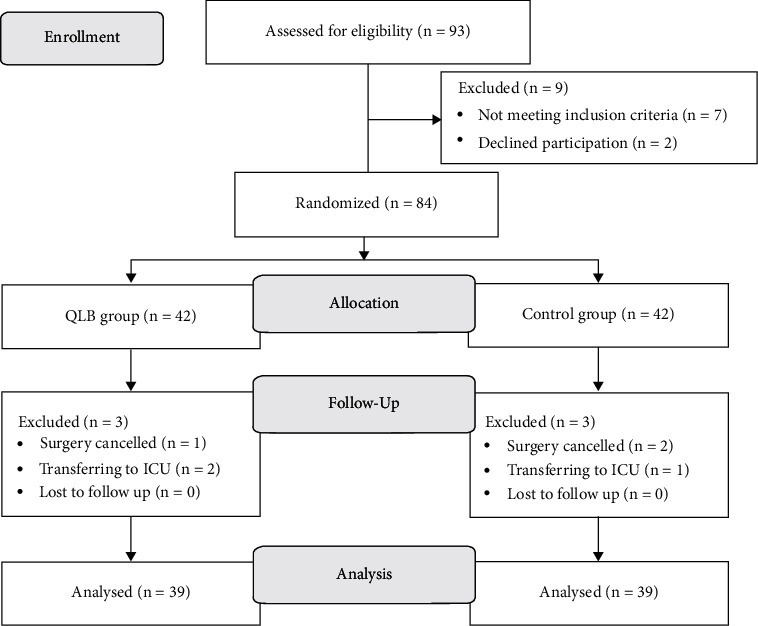
CONSORT flow diagram. QLB: quadratus lumborum block.

The clinical trial was conducted from August 2019 to January 2020. The flow diagram for this study according to CONSORT guidelines is shown in Figure 1. A total of 93 participants were enrolled in this study. Seven patients did not meet the inclusion criteria, and 2 patients declined to participate. Thus, 84 patients were randomly allocated into the QLB group and control group. Six patients were excluded from the analysis due to cancelling surgery (*n* = 3:1 in the QBL group, 2 in the control group) or transferring to ICU (*n* = 3:2 in the QBL group, 1 in the control group). Consequently, 78 patients completed the study. The demographics, psychological state, and clinical data are detailed in Table 1. No significant differences in these parameters were found between the groups.

### 3.1. Quality of Recovery

The primary outcome QoR-15 scores are presented in Table 2. The preoperative global QoR-15 scores were different between the groups (*P*=0.010). However, the mean difference was only 3.4 (95% CI: 0.8–6.0), which was significantly lower than the minimal clinically important difference for the QoR-15 scores. Moreover, the four dimensions, namely, physical comfort, physical independence, psychological support, and pain, were not significantly different. The global QoR-15 score at 24 h after surgery was significantly higher in the QLB group than in the control group (mean difference, 16.9; 95% CI: 11.9–21.9). In addition, the QoR-15 scores for the five dimensions were all significantly higher in the QLB group than in the control group. The overall mean differences between the 2 groups were 4.1 (99% CI: 2.2–6.0) in emotional status, 4.8 (99% CI: 2.8–6.9) in physical comfort, 1.4 (99% CI: 0.7–2.2) in psychological support, 3.7 (99% CI: 2.5–4.9) in physical independence, and 2.1 (99% CI: 1.4–2.9) for pain dimension. The global QoR-15 score at 48 h after surgery was significantly higher in the QLB group than in the control group (mean difference, 10.1; 95% CI: 5.1–15.0). Moreover, physical independence (mean difference, 2.6; 99% CI: 1.1–4.1), emotional status (mean difference, 3.9; 99% CI: 2.0–5.7), and pain (mean difference, 1.0; 99% CI: 0.2–1.8) were improved in the QLB group. However, there were no significant differences between the groups in physical comfort (mean difference, 2.2; 99% CI: −0.4–4.5) and psychological support (mean difference, 0.3; 99% CI: −0.3–0.8).

### 3.2. Intraoperative and Postoperative Opioid Consumption

The cumulative oxycodone consumption was significantly lower in the QLB group than in the control group at 24 h (16.5 ± 6.1 mg vs. 27.5 ± 8.9 mg, *P* < 0.001) and 48 h (28.0 ± 8.0 mg vs. 41.2 ± 10.9 mg, *P* < 0.001) after surgery. In intergroup comparisons at each time interval, opioid consumption was significantly lower in the QLB group during 0–6 h (6.1 ± 2.7 mg vs. 10.5 ± 4.8 mg, *P* < 0.001), 6–24 h (10.4 ± 5.0 mg vs. 17.1 ± 6.7 mg, *P* < 0.001), and 24–48 h (11.4 ± 4.0 mg vs. 13.7 ± 5.8 mg, *P*=0.045) after surgery (Table 3). However, the intraoperative remifentanil consumption was not significantly different between the groups (829.6 ± 361.4 *μ*g vs. 990.9 ± 396.1 *μ*g, *P*=0.143). There was no significant difference in the length of hospital stay between the groups (18.1 ± 5.4 vs. 18.3 ± 5.7, *P*=0.840), but the time to leave the bed in the QLB group was shorter than in the control group (44.0 ± 12.9 h vs. 57.7 ± 17.6 h, *P* < 0.01; Table 4).

### 3.3. Postoperative Pain

**Figure 2:**
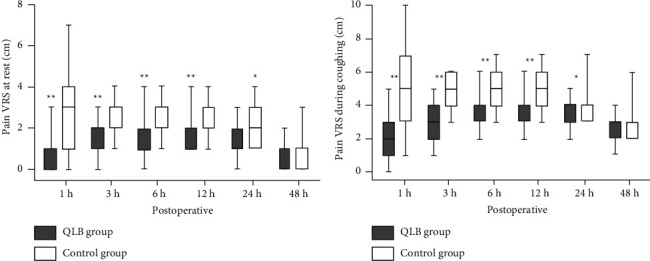
Pain at rest and during coughing at 48 h after surgery: (a) VRS at rest and (b) NRS during coughing. Data are presented as median and interquartile range at rest or during coughing. The VRS scores were significantly lower at 1, 3, 6, 12, and 24 h after surgery in the QLB group than that in the control group. ^*∗*^*P* < 0.05 and ^*∗∗*^*P* < 0.001. QLB: quadratus lumborum block.

**Figure 3:**
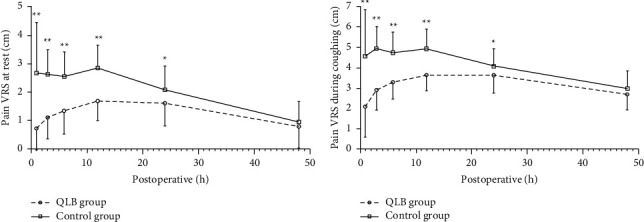
(a) AUC of pain VRS over time at rest, *P* < 0.001 and (b) AUC of pain VRS over time during coughing, *P* < 0.001. Data are presented as mean ± standard deviation. QLB: quadratus lumborum block, AUC: area under the curve, and VRS: verbal rating scale.

The VRS scores at rest (Figure 2(a)) or during coughing (Figure 2(b)) were significantly lower at 1, 3, 6, 12, and 24 h postoperatively in the QLB group compared with the control group (*P* < 0.05). Up to 48 h postoperatively, there were no significant differences between groups at rest (*P*=0.365) and during coughing (*P*=0.131). The areas under the curve (AUCs) of pain VRS versus time at rest (63.3 ± 36.4 mm·h^−1^ vs. 95.2 ± 38.9 mm·h^−1^, *P* < 0.001; Figure 3(a)) and during coughing (153.7 ± 39.8 mm·h^−1^ vs. 190.8 ± 44.5 mm·h^−1^, *P* < 0.001; Figure 3(b)) were significantly smaller in the QLB group than in the control group.

The incidences of CPSP at 3 months in QLB and control groups were 41.0% and 35.5%, respectively. There were no significant differences between the groups (*P*=0.817). Although the incidence of CPSP at 6 months was lower in the QLB group than in the control group (12.8% vs. 25.6%, *P*=0.151), no significant difference between the groups was observed (Table 4).

### 3.4. Adverse Effects

Postoperative nausea occurred in 10 (25.6%) patients in the QLB group and 20 (51.3%) patients in the control group, and a significant difference was found between the groups (*P*=0.035). Postoperative vomiting occurred in 2 (5.1%) patients in the QLB group and 7 (17.9%) patients in the control group, but no significant difference was observed between the groups (*P*=0.154). In addition, there were no significant differences in postoperative dizziness (*P*=0.135) or pruritus (*P*=0.358; Table 4).

## 4. Discussion

The current study demonstrated that compared with general anesthesia alone, the combination of QLB significantly improved the QoR in patients following open gastrointestinal surgery. Furthermore, our results revealed that preoperative ultrasound-guided bilateral QLB had an advantage in relieving pain in the early postoperative period with a smaller burden of pain over time (AUC of VRS) at rest and during coughing, reduced the cumulative analgesic consumption, induced an earlier time to leave the bed, and lowered the incidence of nausea. However, the incidence of CPSP was not significantly reduced by QLB. These findings suggest that preoperative QLB may be an effective regional block to promote rapid recovery after gastrointestinal surgery.

QLB is a relatively new fascial plane block technique that includes different block types. An anatomic study demonstrated that posteromedial QLB could provide better cranial spread than posterolateral QLB. The total extent of the injectate distribution in posteromedial QLB was similar to that in the low thoracic erector spinae plane block [37]. Therefore, in this study, we implemented posteromedial QLB approaches to determine whether QLB could improve postoperative QoR in patients undergoing gastrointestinal surgery.

This is the first study to evaluate the efficacy of QLB on QoR after gastrointestinal surgery. The results revealed that the global QoR-15 score at 24 h after surgery was significantly higher in the QLB group. Meanwhile, higher QoR-15 scores for the five dimensions and higher global QoR-15 scores at 48 h after surgery were observed in the QLB group. Moreover, three of these dimensions, namely, physical independence, emotional status, and pain, were significantly improved in the QLB group compared with the control group. However, there were no significant differences in physical comfort and psychological support between the groups.

Wang et al. [38] reported that lateral QLB could enhance recovery after laparoscopic colorectal surgery where the QoR-15 score at 48 h postoperatively was greatly improved. However, the study did not investigate the five dimensions of the QoR-15 and the score at 24 h after surgery. Another randomized controlled trial evaluated whether epidural block combined with general anesthesia improved the QoR-15 scores in patients undergoing laparoscopic radical resection of colorectal cancer [4]. The study demonstrated that the global QoR-15 scores at 24 and 48 h after surgery were significantly improved by epidural block. Meanwhile, all five dimensions of the QoR-15 at 24 h after surgery, physical comfort, and pain at 48 h after surgery were also improved compared with the control group. Kim et al. [39] reported that in patients undergoing video-assisted thoracic surgery, those who received single-injection superficial serratus plane block got higher QoR-40 scores on postoperative days 1 and 2. However, three dimensions including psychological support, emotional status, and pain on postoperative day 1 and psychological support and pain on postoperative day 2 did not improve.

On the contrary, Kawk et al. [18] demonstrated that lateral QLB could not improve the global QoR-15 scores at 48 h after surgery in patients undergoing laparoscopic nephrectomy. This may be explained by the small sample size, which could not detect differences in the QoR. Moreover, this study did not analyze the efficacy of QLB 24 h after surgery. Another randomized controlled trial demonstrated that single-shot posterior QLB did not improve the QoR at 24 h after major gynecological laparoscopic surgery [40], possibly because QLB failed to provide any additional benefit under postoperative multimodal analgesia.

A meta-analysis demonstrated that QLB is applicable in abdominal or hip surgery patients because it can reduce postoperative opioid consumption and pain intensity [41]. Another meta-analysis showed that QLB had a better postoperative analgesic effect than placebo after abdominal wall and hip surgeries. However, the advantages were less pronounced than other analgesic techniques [42]. Wang et al. [38] assessed the efficacy of lateral QLB on postoperative pain in patients undergoing laparoscopic colorectal surgery. They reported that QLB relieved pain intensity during coughing at 2, 6, and 12 h after surgery, while the efficacy at rest only occurred at 2 and 6 h after surgery. Additionally, postoperative cumulative sufentanil consumption during 0–6, 6–12, and 12–24 h were significantly reduced in the QLB group. Meouchy et al. [43] demonstrated the efficacy of QLB on postoperative analgesia in abdominoplasty. The results showed that the QLB could noticeably decrease pain NRS scores at rest or with effort. The cumulative tramadol consumption was significantly reduced in the QLB group in the first 48 h after surgery. Similarly, our results revealed that the QLB led to less cumulative oxycodone consumption during 0–6, 6–24, and 24–48 h and lower pain VRS scores at rest or during coughing in the first 24 h after surgery. Furthermore, smaller areas under the pain VRS versus time curves (AUCs) at rest and during coughing were observed in the QLB group in the first 48 h after surgery. This may stem from the preemptive analgesic effect of regional blocks that can prevent hyperpathia by inhibiting peripheral and central sensitization.

In contrast to the results of our study, Boulianne et al. [44] revealed that QL2 block could not decrease opioid consumption or relieve pain at 24 h after colorectal resection. Nevertheless, as part of a routine multimodal analgesic regimen, local wound infiltration with 10 ml 2% of xylocaine could also reduce postoperative pain intensity, with local wound infiltration possibly concealing the analgesic effect of QLB. In addition, Irwin et al. [45] assessed the efficacy of QLB as part of a multimodal analgesic regimen in patients undergoing caesarean section and demonstrated that intrathecal morphine may mask the effect of QLB on postoperative pain.

It is widely believed that QLB can effectively reduce postoperative opioid consumption. However, the benefit of QLB in sparing intraoperative opioid use remains controversial. Fujimoto et al. [40] reported that posterior QLB significantly reduced intraoperative remifentanil consumption in patients undergoing major laparoscopic gynecological surgery. Contrarily, a randomized controlled study of patients undergoing laparoscopic colorectal surgery showed that lateral QLB could not reduce intraoperative remifentanil consumption but intraoperative sufentanil consumption [38]. Nevertheless, we did not observe a significant difference in intraoperative remifentanil consumption between QLB and control groups. Similarly, Boulianne et al. [44] reported that posterior QBL could not spare the intraoperative dose of remifentanil in colorectal resection.

A meta-analysis revealed that QLB could reduce the incidence of postoperative nausea and vomiting (PONV) in patients undergoing abdominal or hip surgery. Korgvee et al. [41] demonstrated that QLB was associated with a significantly lower NRS score of nausea at 24 h after laparoscopic gynecologic surgery. In contrast, a higher incidence of nausea may have been caused by continuously more incredible pain and more analgesia consumption than the control group. We also found that QLB diminished the incidence of nausea, which tends to be an opioid-related side effect. The incidence of postoperative vomiting was lower in the QLB group, but there was no significant difference between the groups. One possible reason may have been that the sample size was relatively small, as it was not calculated according to the incidence of PONV. Furthermore, a randomized controlled trial found that posterior QBL could not reduce the incidence of PONV in patients undergoing colorectal resection [44]. As the study did not reveal a reduction in postoperative morphine consumption when QLB was applied, the incidence of opioid-related side effects, such as PONV, may not decrease.

Regional anesthesia provides superior APSP management, but its use remains controversial in preventing the development of CPSP. Moderate-quality evidence has confirmed the efficacy of epidural anesthesia for the prevention of CPSP in patients undergoing open thoracotomy. In contrast, low-quality evidence has demonstrated the effectiveness of paravertebral block for preventing CPSP in patients undergoing breast cancer surgery [46, 47]. However, regional anesthesia has not been proven to reduce the risk of CPSP in other procedures, such as limb amputation, hernia repair, cardiac surgery, or abdominal surgery. Our study did not find QLB efficacious in reducing the risk of developing chronic pain at 3 and 6 months after surgery. These findings are consistent with a retrospective cohort study, which demonstrated that TAP block could not reduce the incidence of CPSP at 3 and 6 months after elective colorectal surgery [48]. Nevertheless, in a randomized double-blinded trial, epidural anesthesia combined with continuous intravenous ketamine could reduce the incidence of chronic pain after major digestive surgery [49]. Therefore, a multimodal analgesic regimen should be recommended to reduce the risk of developing CPSP.

Preoperative chronic pain [50] and psychological disorders, such as anxiety [51], depression [52], and expected postsurgical pain [33], were independently associated with postoperative pain. A previous study has reported that postoperative pain is strongly associated with the early QoR after surgery [3]. We analyzed the data on preoperative chronic pain, anxiety, depression, and expected postsurgical pain; however, there were no significant differences between the groups. Thus, we further minimized the risk of bias by adjusting for confounding factors, which is also an innovation of our study.

Our study had several limitations. First, we did not confirm the effectiveness of QLB by sensory level testing after local anesthetic injection after considering group allocation blinding. Instead, we observed the spread patterns of the local anesthetic by real-time ultrasound. Second, we found that the groups' preoperative global QoR-15 scores and emotional status scores differed. However, the mean differences were only 3.4 and 2.1, respectively.

The preoperative psychological risks, such as anxiety, depression, and expected postsurgical pain, were not significantly different. Third, we did not investigate short- and long-term time postoperative time points because previous studies have demonstrated that regional anesthesia only improves the QoR on postoperative days 1 to 2 [4, 38, 53, 54]. Fourth, the sample size may have been too small to yield valid results in secondary outcomes. Thus, further studies are needed to investigate whether QLB can reduce postoperative opioid consumption, acute pain intensity, and CPSP and PONV incidence. Finally, we used one specific type of QLB that has been widely described in the literature [21, 37]. Therefore, our results cannot be generalized to other types of QLB.

## 5. Conclusions

In conclusion, single-injection posteromedial QLB in patients undergoing gastrointestinal surgery enhances the QoR and reduces acute pain intensity and opioid consumption during the early postoperative period. However, the incidences of chronic pain and vomiting were not significantly decreased. Further studies are needed to support the application of QLB in the management of chronic pain.

## Figures and Tables

**Table 1:** Demographic, psychological, and clinical characteristics of the patients.

Variables	QLB group, *N* = 39	Control group, *N* = 39	Statistical value	*P* value
Age, years	66.2 ± 8.6	63.4 ± 11.1	−1.234	0.221
Male, *n* (%)	24 (61.5%)	23 (56.4%)	0.212	0.818
BMI, kg/m^2^	23.2 ± 3.3	23.3 ± 3.6	0.086	0.932
Hypertension, *n* (%)	19 (48.7%)	22 (56.4%)	0.463	0.650
Diabetes, *n* (%)	3 (7.7%)	7 (17.9%)	1.835	0.310
Preoperative chronic pain	10 (25.6%)	12 (30.8%)	0.253	0.802
ASA physical status, *n* (%)			3.174	0.242
ASA I	1 (2.6%)	4 (10.3%)		
ASA II	35 (89.7%)	29 (74.3%)		
ASA III	3 (7.7%)	6 (15.4%)		
Site of surgery			3.303	0.238
Stomach	15 (38.5%)	23 (59.0%)		
Colon	17 (43.6%)	11 (28.2%)		
Rectum	7 (17.9%)	5 (12.8%)		
Duration of surgery, min	170.9 ± 47.9	178.1 ± 55.8	0.609	0.544
Duration of anesthesia, min	205.3 ± 48.5	198.7 ± 60.1	−0.529	0.598
Blood loss volume, ml	200.0 (100.0–225.0)	200.0 (100.0–225.0)	754.000	0.949
Indwelling tube time, day	8.0 ± 2.6	8.6 ± 3.3	0.954	0.343
HADS: anxiety	1.0 (0.0–3.5)	3.0 (0.5–7.0)	581.500	0.070
HADS: depression	1.0 (1.0–4.5)	2.0 (1.0–6.5)	573.000	0.056
Expected postsurgical pain	5.0 (3.0–6.0)	5.0 (5.0–7.0)	567.500	0.051

Data are presented as mean ± standard deviation, median (interquartile range), or number (percentage). QLB: quadratus lumborum block, BMI: body mass index, ASA: American Society of Anesthesiologists, and HADS: hospital anxiety and depression scale.

**Table 2:** QoR-15 questionnaire global and dimension scores.

Variables	QLB group, *N* = 39	Control group, *N* = 39	Statistical value	*P* value
Before surgery				
Physical comfort	48.6 ± 1.8	47.6 ± 3.0	−1.919	0.059
Physical independence	19.7 ± 0.7	19.6 ± 1.1	−0.710	0.480
Psychological support	19.9 ± 0.2	19.9 ± 0.2	−0.582	0.562
Emotional status	38.2 ± 2.9	36.1 ± 4.3	−2.478	0.015
Pain	20.0 ± 0.0	19.9 ± 0.5	−1.000	0.320
Global QoR-15	146.6 ± 4.1	143.1 ± 7.1	−2.631	0.010

24 h after surgery				
Physical comfort	43.6 ± 3.2	38.8 ± 5.6	−4.687	<0.001
Physical independence	16.0 ± 2.2	12.3 ± 3.0	−6.281	<0.001
Psychological support	19.5 ± 1.0	18.1 ± 2.2	−3.735	<0.001
Emotional status	36.7 ± 3.6	32.6 ± 4.8	−4.249	<0.001
Pain	17.9 ± 1.5	15.7 ± 1.8	−3.925	<0.001
Global QoR-15	134.4 ± 7.8	117.5 ± 13.5	−5.537	<0.001

48 h after surgery				
Physical comfort	45.2 ± 3.3	43.0 ± 4.5	−1.981	0.054
Physical independence	17.7 ± 1.9	15.1 ± 3.3	−3.389	0.001
Psychological support	19.6 ± 0.8	19.4 ± 1.2	−0.868	0.390
Emotional status	38.8 ± 1.6	35.0 ± 4.4	−4.213	<0.001
Pain	17.8 ± 1.8	18.8 ± 1.0	−2.491	0.016
Global QoR-15	140.3 ± 5.8	130.2 ± 10.8	−4.062	<0.001

Data are presented as mean ± standard deviation. QoR-15: 15-item quality of recovery.

**Table 3:** Cumulative oxycodone consumption at 48 h after surgery.

Oxycodone consumption, mg	QLB group, *N* = 39	Control group, *N* = 39	Statistical value	*P* value
0–6 h	6.1 ± 2.7	10.5 ± 4.8	4.972	<0.001
6–24 h	10.4 ± 5.0	17.1 ± 6.7	4.987	<0.001
24–48 h	11.4 ± 4.0	13.7 ± 5.8	2.035	0.045
0–24 h	16.5 ± 6.1	27.5 ± 8.9	6.340	<0.001
0–48 h	28.0 ± 8.0	41.2 ± 10.9	6.163	<0.001

Data are presented as mean ± standard deviation. QLB: quadratus lumborum block.

**Table 4:** Outcome measurements of the patients.

Variables	QLB group, *N* = 39	Control group, *N* = 39	Statistical value	*P* value
Remifentanil consumption, *μ*g	829.6 ± 361.4	990.9 ± 396.1	1.488	0.143
Time to leave bed, hours	44.0 ± 12.9	57.7 ± 17.6	3.935	<0.001
Length of hospital stay, days	18.1 ± 5.4	18.3 ± 5.7	0.203	0.840
Adverse effects				
Nausea, *n* (%)	10 (25.6%)	20 (51.3%)	5.417	0.035
Vomiting, *n* (%)	2 (5.1%)	7 (17.9%)	3.140	0.154
Dizziness, *n* (%)	8 (20.5%)	15 (38.5%)	3.021	0.135
Pruritus, *n* (%)	1 (2.5%)	4 (10.3%)	1.923	0.358
Incidence of CPSP				
3 months, *n* (%)	16 (41.0%)	15 (35.5%)	0.054	0.817
6 months, *n* (%)	5 (12.8%)	10 (25.6%)	2.063	0.151

Data are presented as mean ± standard deviation, median (interquartile range), or number (percentage). QLB: quadratus lumborum block and CPSP: chronic postsurgical pain.

## Data Availability

The data used to support the findings of this study are available from the corresponding author upon request.

## References

[1] Siegel R. L., Miller K. D., Fuchs H. E., Jemal A. (2021). Cancer statistics, 2021. *CA: A Cancer Journal of Clinicians*.

[2] Joshi G. P., Bonnet F., Kehlet H. (2013). Evidence-based postoperative pain management after laparoscopic colorectal surgery. *Colorectal Disease: The Official Journal of the Association of Coloproctology of Great Britain and Ireland*.

[3] Wu C. L., Rowlingson A. J., Partin A. W. (2005). Correlation of postoperative pain to quality of recovery in the immediate postoperative period. *Regional Anesthesia and Pain Medicine*.

[4] Liu Q., Lin J.-Y., Zhang Y.-F. (2020). Effects of epidural combined with general anesthesia versus general anesthesia on quality of recovery of elderly patients undergoing laparoscopic radical resection of colorectal cancer: a prospective randomized trial. *Journal of Clinical Anesthesia*.

[5] Sakamoto B., Harker G., Eppstein A. C., Gwirtz K. (2016). Efficacy of local anesthetic with dexamethasone on the quality of recovery following total extraperitoneal bilateral inguinal hernia repair: a randomized clinical trial. *JAMA surgery*.

[6] Ding W., Li W., Zeng X., Li J., Jiang J., Guo C. (2017). Effect of adding dexmedetomidine to ropivacaine on ultrasound-guided dual transversus abdominis plane block after gastrectomy. *Journal of Gastrointestinal Surgery*.

[7] Oh T. K., Yim J., Kim J. (2017). Effects of preoperative ultrasound-guided transversus abdominis plane block on pain after laparoscopic surgery for colorectal cancer: a double-blind randomized controlled trial. *Surgical Endoscopy*.

[8] Blanco R., Ansari T., Riad W., Shetty N. (2016). Quadratus lumborum block versus transversus abdominis plane block for postoperative pain after cesarean delivery: a randomized controlled trial. *Regional Anesthesia and Pain Medicine*.

[9] Elsharkawy H., El-Boghdadly K., Barnes T. J. (2019). The supra-iliac anterior quadratus lumborum block: a cadaveric study and case series. *Canadian Journal of Anesthesia*.

[10] Kukreja P., MacBeth L., Sturdivant A. (2019). Anterior quadratus lumborum block analgesia for total hip arthroplasty: a randomized, controlled study. *Regional Anesthesia and Pain Medicine*.

[11] Aksu C., Gürkan Y. (2018). Ultrasound guided quadratus lumborum block for postoperative analgesia in pediatric ambulatory inguinal hernia repair. *Journal of Clinical Anesthesia*.

[12] Öksüz G., Arslan M., Urfalıoğlu A. (2020). Comparison of quadratus lumborum block and caudal block for postoperative analgesia in pediatric patients undergoing inguinal hernia repair and orchiopexy surgeries: a randomized controlled trial. *Regional Anesthesia and Pain Medicine*.

[13] Ökmen K., Metin Ökmen B., Topal S. (2018). Ultrasound-guided posterior quadratus lumborum block for postoperative pain after laparoscopic cholecystectomy: a randomized controlled double blind study. *Journal of Clinical Anesthesia*.

[14] Baytar Ç., Yılmaz C., Karasu D., Topal S. (2019). Comparison of ultrasound-guided subcostal transversus abdominis plane block and quadratus lumborum block in laparoscopic cholecystectomy: a prospective, randomized, controlled clinical study. *Pain research & management*.

[15] Krohg A., Ullensvang K., Rosseland L. A., Langesæter E., Sauter A. R. (2018). The analgesic effect of ultrasound-guided quadratus lumborum block after cesarean delivery: a randomized clinical trial. *Anesthesia & Analgesia*.

[16] Ishio J., Komasawa N., Kido H., Minami T. (2017). Evaluation of ultrasound-guided posterior quadratus lumborum block for postoperative analgesia after laparoscopic gynecologic surgery. *Journal of Clinical Anesthesia*.

[17] Dam M., Hansen C. K., Poulsen T. D. (2019). Transmuscular quadratus lumborum block for percutaneous nephrolithotomy reduces opioid consumption and speeds ambulation and discharge from hospital: a single centre randomised controlled trial. *British journal of anaesthesia*.

[18] Kwak K.-H., Baek S. I., Kim J. K., Kim T.-H., Yeo J. (2020). Analgesic effect of ultrasound-guided preoperative unilateral lateral quadratus lumborum block for laparoscopic nephrectomy: a randomized, double-blinded, controlled trial. *Journal of Pain Research*.

[19] Dam M., Hansen C., Poulsen T. D. (2021). Transmuscular quadratus lumborum block reduces opioid consumption and prolongs time to first opioid demand after laparoscopic nephrectomy. *Regional Anesthesia and Pain Medicine*.

[20] Dewinter G., Coppens S., Van de Velde M. (2018). Quadratus lumborum block versus perioperative intravenous lidocaine for postoperative pain control in patients undergoing laparoscopic colorectal surgery: a prospective, randomized, double-blind controlled clinical trial. *Annals of Surgery*.

[21] Huang D., Song L., Li Y., Xu Z., Li X., Li C. (2020). Posteromedial quadratus lumborum block versus transversus abdominal plane block for postoperative analgesia following laparoscopic colorectal surgery: a randomized controlled trial. *Journal of Clinical Anesthesia*.

[22] Xia Q., Ding W., Lin C., Xia J., Xu Y., Jia M. (2021). Postoperative pain treatment with transmuscular quadratus lumborum block and fascia iliaca compartment block in patients undergoing total hip arthroplasty: a randomized controlled trial. *BMC Anesthesiology*.

[23] Xulei C., Xu L., Minna L. (2020). Ultrasound-guided transmuscular quadratus lumbar block reduces opioid consumption after laparoscopic partial nephrectomy. *Chinese Medical Sciences Journal*.

[24] Stark P. A., Myles P. S., Burke J. A. (2013). Development and psychometric evaluation of a postoperative quality of recovery score. *Anesthesiology*.

[25] Chazapis M., Walker E. M., Rooms M. A., Kamming D., Moonesinghe S. R. (2016). Measuring quality of recovery-15 after day case surgery. *British journal of anaesthesia*.

[26] Finnerty D. T., McMahon A., McNamara J. R., Hartigan S. D., Griffin M., Buggy D. J. (2020). Comparing erector spinae plane block with serratus anterior plane block for minimally invasive thoracic surgery: a randomised clinical trial. *British journal of anaesthesia*.

[27] Lu J., Wang J.-F., Guo C.-L., Yin Q., Cheng W., Qian B. (2021). Intravenously injected lidocaine or magnesium improves the quality of early recovery after laparoscopic cholecystectomy: a randomised controlled trial. *European Journal of Anaesthesiology*.

[28] Kleif J., Waage J., Christensen K. B., Gogenur I. (2018). Systematic review of the QoR-15 score, a patient- reported outcome measure measuring quality of recovery after surgery and anaesthesia. *British journal of anaesthesia*.

[29] Demumieux F., Ludes P.-O., Diemunsch P. (2020). Validation of the translated quality of recovery-15 questionnaire in a french-speaking population. *British Journal of Anaesthesia*.

[30] Kim D., Kim J. K., Yeo J. (2020). Translation and validation of the korean version of the postoperative quality of recovery score QoR-15. *BioMed Research International*.

[31] Bu X. S., Zhang J., Zuo Y. X. (2016). Validation of the Chinese version of the quality of recovery-15 score and its comparison with the post-operative quality recovery scale. *The Patient—Patient-Centered Outcomes Research*.

[32] Ferreira-Valente M. A., Pais-Ribeiro J. L., Jensen M. P. (2011). Validity of four pain intensity rating scales. *Pain*.

[33] Pan P. H., Tonidandel A. M., Aschenbrenner C. A., Houle T. T., Harris L. C., Eisenach J. C. (2013). Predicting acute pain after cesarean delivery using three simple questions. *Anesthesiology*.

[34] Zigmond A. S., Snaith R. P. (1983). The hospital anxiety and depression scale. *Acta Psychiatrica Scandinavica*.

[35] Myles P. S., Myles D. B., Galagher W., Chew C., MacDonald N., Dennis A. (2016). Minimal clinically important difference for three quality of recovery scales. *Anesthesiology*.

[36] Myles P. S., Myles D. B. (2021). An updated minimal clinically important difference for the QoR-15 scale. *Anesthesiology*.

[37] Elsharkawy H., Bajracharya G. R., El-Boghdadly K., Drake R. L., Mariano E. R. (2019). Comparing two posterior quadratus lumborum block approaches with low thoracic erector spinae plane block: an anatomic study. *Regional Anesthesia and Pain Medicine*.

[38] Wang D., He Y., Chen X., Lin Y., Liu Y., Yu Z. (2021). Ultrasound guided lateral quadratus lumborum block enhanced recovery in patients undergoing laparoscopic colorectal surgery. *Advances in Medical Sciences*.

[39] Kim D. H., Oh Y. J., Lee J. G., Ha D. H., Chang Y. J., Kwak H. J. (2018). Efficacy of ultrasound-guided serratus plane block on postoperative quality of recovery and analgesia after video-assisted thoracic surgery: a randomized, triple-blind, placebo-controlled study. *Anesthesia & Analgesia*.

[40] Fujimoto H., Irie T., Mihara T., Mizuno Y., Nomura T., Goto T. (2019). Effect of posterior quadratus lumborum blockade on the quality of recovery after major gynaecological laparoscopic surgery: a randomized controlled trial. *Anaesthesia & Intensive Care*.

[41] Korgvee A., Junttila E., Koskinen H., Huhtala H., Kalliomaki M.-L. (2021). Ultrasound-guided quadratus lumborum block for postoperative analgesia: a systematic review and meta-analysis. *European Journal of Anaesthesiology*.

[42] Uppal V., Retter S., Kehoe E., McKeen D. M. (2020). Quadratus lumborum block for postoperative analgesia: a systematic review and meta-analysis. *Canadian Journal of Anesthesia*.

[43] Meouchy M. G., Awaida C. J., Jabbour H. J., Rayess Y. A., Jabbour S. F., Nasr M. W. (2021). Ultrasound-guided quadratus lumborum block for postoperative pain in abdominoplasty: a randomized controlled study. *Plastic and Reconstructive Surgery*.

[44] Boulianne M., Paquet P., Veilleux R. (2020). Effects of quadratus lumborum block regional anesthesia on postoperative pain after colorectal resection: a randomized controlled trial. *Surgical Endoscopy*.

[45] Irwin R., Stanescu S., Buzaianu C. (2020). Quadratus lumborum block for analgesia after caesarean section: a randomised controlled trial. *Anaesthesia*.

[46] Weinstein E. J., Levene J. L., Cohen M. S. (2018). Local anaesthetics and regional anaesthesia versus conventional analgesia for preventing persistent postoperative pain in adults and children. *Cochrane Database of Systematic Reviews*.

[47] Levene J. L., Weinstein E. J., Cohen M. S. (2019). Local anesthetics and regional anesthesia versus conventional analgesia for preventing persistent postoperative pain in adults and children: a cochrane systematic review and meta-analysis update. *Journal of Clinical Anesthesia*.

[48] Pan Z.-Y., Hu Z.-H., Zhang F., Xie W.-X., Tang Y.-Z., Liao Q. (2020). The effect of transversus abdominis plane block on the chronic pain after colorectal surgery: a retrospective cohort study. *BMC Anesthesiology*.

[49] Lavand’homme P., De Kock M., Waterloos H. (2005). Intraoperative epidural analgesia combined with ketamine provides effective preventive analgesia in patients undergoing major digestive surgery. *Anesthesiology*.

[50] Gerbershagen H. J., Pogatzki-Zahn E., Aduckathil S. (2014). Procedure-specific risk factor analysis for the development of severe postoperative pain. *Anesthesiology*.

[51] Ip H. Y. V., Abrishami A., Peng P. W. H., Wong J., Chung F. (2009). Predictors of postoperative pain and analgesic consumption. *Anesthesiology*.

[52] Yang M. M. H., Hartley R. L., Leung A. A. (2019). Preoperative predictors of poor acute postoperative pain control: a systematic review and meta-analysis. *BMJ Open*.

[53] Yao Y., Lin C., He Q., Gao H., Jin L., Zheng X. (2020). Ultrasound-guided bilateral superficial cervical plexus blocks enhance the quality of recovery in patients undergoing thyroid cancer surgery: a randomized controlled trial. *Journal of Clinical Anesthesia*.

[54] Canıtez A., Kozanhan B., Aksoy N., Yildiz M., Tutar M. S. (2021). Effect of erector spinae plane block on the postoperative quality of recovery after laparoscopic cholecystectomy: a prospective double-blind study. *British Journal of Anaesthesia*.

